# Derivation, Comprehensive Analysis, and Assay Validation of a Pyroptosis-Related lncRNA Prognostic Signature in Patients With Ovarian Cancer

**DOI:** 10.3389/fonc.2022.780950

**Published:** 2022-02-24

**Authors:** Xueyan Cao, Qingquan Zhang, Yu Zhu, Xiaoqing Huo, Junze Bao, Min Su

**Affiliations:** ^1^ Department of Obstetrics and Gynecology, Affiliated Hospital of Nantong University, Nantong, China; ^2^ Department of Cardiology, Affiliated Hospital of Nantong University, Nantong, China; ^3^ Medical College, Nantong University, Nantong, China

**Keywords:** lncRNAs, prognostic signature, ovarian cancer, immune infiltration, pyroptosis

## Abstract

**Background:**

Pyroptosis is regulated by long non-coding RNAs (lncRNAs) in ovarian cancer (OC). Therefore, a comprehensive analysis of pyroptosis-related lncRNAs (PRLs) in OC is crucial for developing therapeutic strategies and survival prediction.

**Methods:**

Based on public database raw data, mutations in the landscape of pyroptosis-related genes (PRGs) in patients with OC were investigated thoroughly. PRLs were identified by calculating Pearson correlation coefficients. Cox and LASSO regression analyses were performed on PRLs to screen for lncRNAs participating in the risk signature. Furthermore, receiver operating characteristic (ROC) curves, Kaplan–Meier survival analyses, decision curve analysis (DCA) curves, and calibration curves were used to confirm the clinical benefits. To assess the ability of the risk signature to independently predict prognosis, it was included in a Cox regression analysis with clinicopathological parameters. Two nomograms were constructed to facilitate clinical application. In addition, potential biological functions of the risk signature were investigated using gene function annotation. Subsequently, immune-related landscapes and *BRCA1/2* mutations were compared in different risk groups using diverse bioinformatics algorithms. Finally, we conducted a meta-analysis and *in-vitro* assays on alternative lncRNAs.

**Results:**

A total of 374 patients with OC were randomized into training and validation cohorts (7:3). A total of 250 PRLs were selected from all the lncRNAs. Subsequently, a risk signature (DICER1-AS1, MIR600HG, AC083880.1, AC109322.1, AC007991.4, IL6R-AS1, AL365361.1, and AC022098.2) was constructed to distinguish the risk of patient survival. The ROC curve, K-M analysis, DCA curve, and calibration curve indicated excellent predictive performance for determining overall survival (OS) based on the risk signature in each cohort (*p* < 0.05). The Cox regression analysis indicated that the risk signature was an independent prognostic factor for OS (*p* < 0.05). Moreover, significant differences in the immune response and *BRCA1* mutations were identified in different groups distinguished by the risk signature (*p* < 0.05). Interestingly, *in-vitro* assays showed that an alternative lncRNA (*DICER1-AS1*) could promote OC cell proliferation.

**Conclusion:**

The PRL risk signature could independently predict overall survival and guide treatment in patients with OC.

## 1. Introduction

Ovarian cancer (OC) is the most common cause of death from malignant tumors of the female reproductive system and has a high recurrence and mortality. A total of 80% of OC patients are already at an advanced stage when they are diagnosed due to a lack of efficient early screening strategies (1). As current therapies have not improved survival rates, novel targets are required to improve the clinical status of patients with ovarian cancer. Hence, new prognostic models are urgently required.

Gesdermin-d (GSDMD)-mediated programmed necrotic cell death is thought to be pyroptosis, also known as cellular inflammatory necrosis (2). Cleavage of GSDMD, which is downstream of caspase, and activation of dormant cytokines are required for pyroptosis, which is triggered by specific inflammatory vesicles (3). The relationship between pyroptosis and cancer is complex. Although pyroptosis inhibits tumor growth and development, it also produces a microenvironment that promotes cancer growth (4). The influence of pyroptosis on tumor cell growth, invasion, and metastasis, and hence, on cancer prognosis, has been increasingly investigated (5–7). Long non-coding RNAs (lncRNAs) are defined as non-protein-coding transcripts that are longer than 200 nucleotides (8). lncRNAs have been shown to play major regulatory roles in various diseases, including ovarian cancer (9). It is interesting to note that lncRNAs are mediators of pyroptosis in cancer (10). However, the clinical significance of most lncRNAs, particularly PRLs, has not been clearly studied.

A new pyroptosis-related gene (PRGs) signature was found to influence ovarian cancer prognosis in a recent study (11). In addition, many lncRNA signatures have been developed for OC patients, such as a risk score system based on co-expression network analysis (12), prognosis-associated lncRNAs as biomarkers (13), and prognostic lncRNA biomarkers based on the ceRNA network (14). However, the above study only focused on the predictive value of 33 PRGs rather than covering upstream lncRNAs. The purpose of our study was to use bioinformatics to explore the prognostic value of pyroptosis-related lncRNAs (PRLs) and the landscape of PRG mutations. Preliminary experimental validation was performed using an alternative lncRNA. These findings may contribute to the development of predictive biomarkers and therapeutic targets for OC treatment.

## 2. Materials and Methods

### 2.1 Bioinformatics Datasets and Data Preprocessing

OC clinical data (*n* = 587), OC RNA sequencing profiles (*n* = 379), and normal ovarian epithelial tissue RNA sequencing profiles (*n* = 88) were obtained from the Cancer Genome Atlas (TCGA) (15) and GTEx databases (16). We excluded patients with OC without RNA sequencing and survival time data, and finally, only 374 patients were retained for subsequent analysis. At a ratio of 3:7, the OC patients were divided into two sets (the training cohort and testing cohort) using the “caret” package in R software (17). Meanwhile, lncRNAs and protein-coding genes were identified based on annotation documents from the GENCODE database (18). Additionally, 33 PRGs were extracted based on previous studies (9).

### 2.2 Analysis of Copy Number Variation and Mutations

Mutation and copy number variation (CNV) data, including 436 OC patient samples, were downloaded from the TCGA database. In addition, the mutation frequencies of the 33 PRGs in patients with OC were generated using the “maftools” package (19). The locations of CNV alterations in the 33 PRGs on the 23 chromosomes were plotted using the RCircos package.

### 2.3 Construction of a Prognostic Signature and Two Nomograms Based on PRLs

Prognostic lncRNAs (*p* < 0.05) were screened using a univariate Cox regression analysis. These prognostic lncRNAs were further incorporated into multivariate Cox and LASSO regression analyses to identify the lncRNAs involved in signature construction (20). We used the appropriate *λ* to build the model and control for the complexity of the LASSO regression. The risk score was calculated as follows: risk score for OS =


$$\sum_{i=1}^{\text{n}}{\text{Coef}}_{\text{i}}*{\text{x}}_{\text{i}}$$

For clinical utility, the comparative value (2^−Δct^) was calculated from RT-qPCR results and used for score calculation in our hospital cohort, and the score was further standardized and simplified to generate a risk score (21). The risk score was calculated as follows: Risk score (RT-qPCR) = (Score − Min)/Max.

### 2.4 Exploring the Clinical Benefits of Prognostic Signatures

The risk score for each OC patient was summed using the above formula. Risk signatures for predicting survival were assessed using AUC and decision curve analysis (DCA) curves (22). We calculated the risk score of each patient by determining their cutoff value using the receiver operating characteristic (ROC) curve, which is used to select the “high-risk” and “low-risk” groups. The Kaplan–Meier survival analysis and log-rank test suggested that the high-risk groups had shorter survival times than the low-risk groups.

### 2.5 Functional Enrichment and Differential Expression Analysis

A network of PRLs with co-expressed genes was constructed using the Cytoscape software (23). Gene enrichment analysis was performed on differentially expressed ARGs using related packages in R software. Meanwhile, we used the “limma” package to explore the differential expression of lncRNAs participating in PRL signatures between normal samples (GTEx) and OC samples (TCGA) (24).

### 2.6 Immune Infiltration Analysis

To explore the differences in immune cell infiltration, we simultaneously used six algorithms [TIMER (25), CIBERSORT (26), quanTIseq (27), MCP-counter (28), xCell (29), and EPIC (30)] to estimate the abundance of immune cells in the different risk groups distinguished by the PRL signature. Moreover, we used the ssGSEA algorithm to quantify immune functions and pathways between the low- and high-risk groups (31). More importantly, we explored the expression levels of immune checkpoint-related genes in the different risk groups.

### 2.7 *In-Vitro* Assays

In this study, we used cell culture, transfection, CCK-8, and qRT-PCR for *in-vitro* assays. The Shanghai Cell Institute Country Cell Bank provided the cell lines SKOV-3, A2780, HO-8910PM, and IOSE80. GenePharma generated and annealed small interfering RNA (si-RNA-1/2/3) oligos for *DICER-AS1* and a general negative control. Following the manufacturer’s instructions, each siRNA duplex was transfected into cells using Lipofectamine^®^ 2000 (Invitrogen, Carlsbad, CA, USA). A2780 and SKOV-3 cells were seeded in 96-well plates, with or without *DICER-AS1* knockdown. After 0, 1, 2, 3, and 4 days of cell culture, cell viability was determined using the CCK-8 assay (Dojindo, Tokyo, Japan). In addition, the *DICER-AS1* primers were as follows: *DICER-AS1-F*, 5′-CGAAGAAATGGAATAACTTCCAAC-3′; *DICER-AS1-R*: 5′-TTGGTCCAAACACAGAAGATC-3′. The details of these methods are provided in (32, 33). The experimental results related to this study were provided by Qingquan Zhang, Nantong University.

### 2.8 Ovarian Cancer Samples

OC tissues were obtained from patients who had undergone surgery at the Affiliated Hospital of Nantong University. In the cohort, 10 pairs of tissues were obtained from patients with OC, between 2017 and 2020. The study was authorized by the Ethical Committee of Affiliated Hospital of Nantong University (2021-K150-01).

### 2.9 Statistical Analysis

All statistical analyses were performed using R software (v.4.0.1). Detailed statistical methods for transcriptome data processing were described in the above section.

## 3 Results

### 3.1 Landscape of Pyroptosis-Related Genes in OC Patients

**Figure 1:**
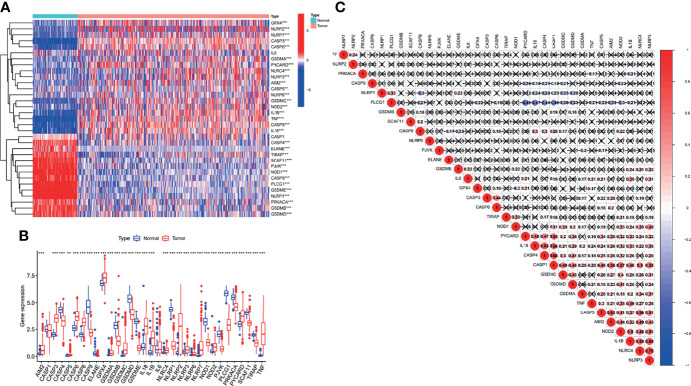
Differential expression and correlation analysis of 33 pyroptosis-related genes (PRGs) in ovarian cancer (OC) patients. The heatmap **(A)** and boxplot **(B)** on the expression of 33 PRGs in OC tissues (red) and normal tissues (blue). The upper and lower ends of the boxes represented the interquartile range of values. **(C)** Correlation analysis of 33 PRGs. **p < 0.01, ***p < 0.001.

**Figure 2:**
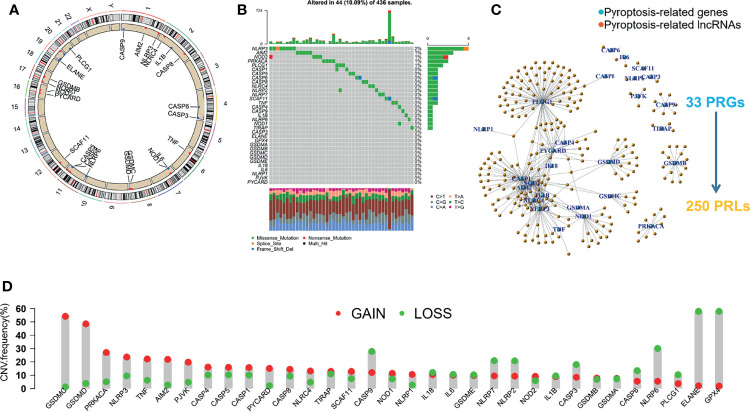
Mutation analysis of PRGs and identification of pyroptosis-related lncRNAs (PRLs) in OC patients. **(A)** The location of CNV alteration of 33 PRGs on 23 chromosomes in the TCGA-OC cohort. **(B)** The mutation frequency and classification of 33 PRGs. **(C)** A network including 33 PRGs and 250 PRLs. **(D)** The CNV variation frequency of 33 PRGs.

First, we explored the expression of 33 PRGs in OC and normal ovarian epithelial tissues based on mRNA expression in the TCGA and GTEx datasets. Interestingly, the heatmap (**Figure 1A**) and boxplot (**Figure 1B**) revealed that all PRGs, except *IL16* and *CASP1*, were aberrantly expressed in OC patients (*p* < 0.05). More specifically, these results may indicate the activation of pyroptosis-related pathways in OC. In addition, we explored the correlations between the 33 PRGs. The results revealed that *NLRP3* showed the strongest positive correlation with *NLRC4* (*r* = 0.75), and *PYCARD* showed the strongest negative correlation with *PLCG1* in patients with OC (*r* = −0.49), as shown in **Figure 1C**. It is also worth noting that we explored the incidence of CNVs and mutations in the 33 PRGs in OC patients from the TCGA database. **Figure 2A** shows the locations of the CNV alterations on the chromosomes for the 33 PRGs. Mutations were present in 44/436 OC samples, and missense mutations were the most common variant classification, as shown in **Figure 2B**. The results also showed that *NLRP3* had the highest mutation frequency among the 33 PRGs. We also investigated the frequency of CNV alterations and found that 33 PRGs showed widespread CNV alterations. Copy number amplification was present in all 33 PRGs except *GPX4* and *ELANE*, with up to 60% amplification in *GSDMC*. Not surprisingly, copy number deletions were also present in most PRGs, with *GPX4* approaching 60% (**Figure 2D**).

Given the significant role of lncRNAs in the regulation of OC mechanisms, we performed a Pearson correlation analysis (|cor| > 0.3, *p* < 0.001) on 13,832 lncRNAs and 33 PRGs from the TCGA gene expression raw data. Ultimately, we screened 250 PRLs for subsequent bioinformatic analysis. The networks are shown in **Figure 2C**.

### 3.2 Construction of a PRL Risk Signature in the Training Cohort

**Figure 3:**
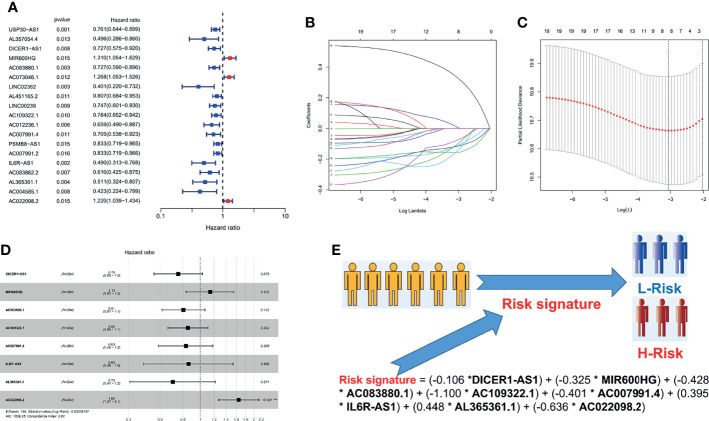
Cox and LASSO regression analyses of 250 PRLs in OC patients. **(A)** The result of univariate Cox regression analysis in 250 PRLs. **(B)** *λ* selection plot. **(C)** LASSO Cox analysis of PRLs. **(D)** A forest plot of eight PRLs participating in signature construction. **(E)** A flowchart of how to distinguish a patient’s risk through a risk signature. *p < 0.05.

A total of 374 OC patients with matched transcriptome data from the TCGA database were downloaded to explore the association between the expression of the above 250 PRLs and OS. To construct and validate the signature, we randomly divided 374 patients with OC into a validation cohort (110 patients) and a training cohort (264 patients). Subsequently, 19 PRLs (*p* < 0.05) were significantly correlated with survival in the univariate Cox regression analysis in the training cohort, as shown in **Figure 3A**. We aimed to avoid the occurrence of collinearity in the high-dimensional transcriptome data; therefore, a LASSO regression analysis (**Figures 3B, C**) was used to further screen eight PRLs, which constituted the prognostic risk signatures of the PRLs. We determined the coefficient of each PRL using the multivariate Cox regression analysis, as shown in **Figure 3D**. Finally, by combining the expression of eight PRLs and the regression coefficients, the risk scores of patients with OC were calculated as follows: risk signature = (−0.106 * DICER1-AS1) + (−0.325 * MIR600HG) + (−0.428 * AC083880.1) + (−1.100 * AC109322.1) + (−0.401 * AC007991.4) + (0.395 * IL6R-AS1) + (0.448 * AL365361.1) + (−0.636 * AC022098.2), as shown in **Figure 3E**.

### 3.3 ROC Analysis and Survival Analysis of the Risk Signatures

**Figure 4:**
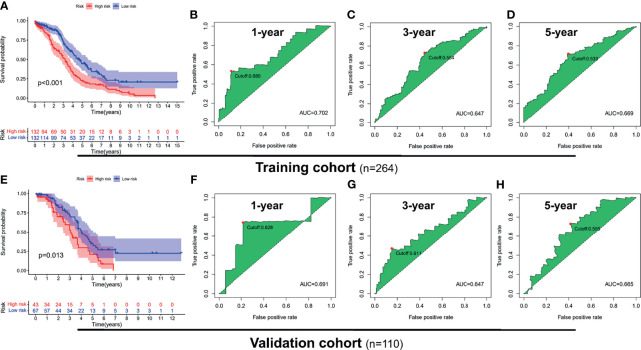
Receiver operating characteristic (ROC) analysis and survival analysis based on risk signature in the training and validation cohorts. Kaplan–Meier survival analysis of the high-risk and low-risk groups in the training cohort **(A)** and validation cohort **(E)**. ROC curve of 1- **(B)**, 3- **(C)**, and 5-year **(D)** survival prediction in the training cohort; ROC curve of 1- **(F)**, 3- **(G)**, and 5-year **(H)** survival prediction in the validation cohort.

**Figure 5:**
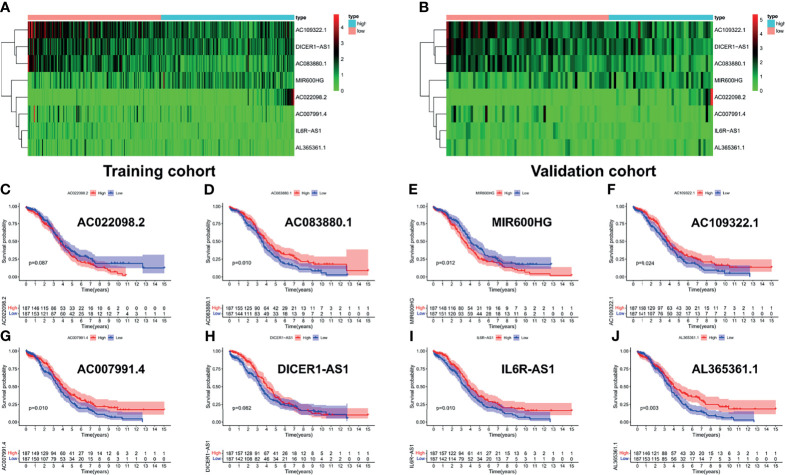
Survival analysis of each lncRNA in risk signature. The expression heatmap of eight PRLs in the training cohort **(A)** and validation cohort **(B)**. Survival analysis of subgroups, including **(C)** AC022098.2, **(D)** AC083880.1, **(E)** MIR600HG, **(F)** AC109322.1, **(G)** AC007991.4, **(H)** DICER1-AS1, **(I)** IL6R-AS1, and **(J)** AL365361.1.

To explore the prognostic value of the PRL signatures, we divided the training and validation cohorts into high- and low-risk groups based on the median values of the risk scores of the training cohort. In each group, the survival times of high-risk patients were significantly shorter than those of low-risk patients (*p* < 0.05), as shown in **Figures 4A, E**. ROC curves demonstrated the predictive effectiveness of the risk signature. The results showed that in the training cohort, the AUCs for predicting 1- (**Figure 4B**), 3- (**Figure 4C**), and 5-year survival (**Figure 4D**) were 0.702, 0.647, and 0.533, respectively. The AUCs for predicting 1- (**Figure 4F**), 3- (**Figure 4G**), and 5-year survival (**Figure 4H**) were 0.691, 0.611, and 0.505 in the validation cohort, respectively. In addition, heatmaps showed the expression of eight PRLs in the different groups (**Figures 5A, B**). In the survival analysis of each lncRNA participating in the risk signature, we found five protective factors, namely, AC083880.1 (**Figure 5D**), AC109322.1 (**Figure 5F**), AC007991.4 (**Figure 5G**), IL6R-AS1 (**Figure 5I**), and AC022098.2 (**Figure 5J**), and only one risky factor, MIR600HG (**Figure 5E**), in the TCGA cohort. Survival analysis of the protective factors showed that PRL overexpression may lead to a longer survival time. In contrast, individuals with upregulated expression of risk factors may have shorter survival times. Although there was no significant difference in survival time among the subgroups of AC022098.2 (**Figure 5C**) and the DICER1-AS1 (**Figure 5H**) group, this is also of concern.

### 3.4 Clinical Correlation Analysis and Independent Prognostic Analysis

**Figure 6:**
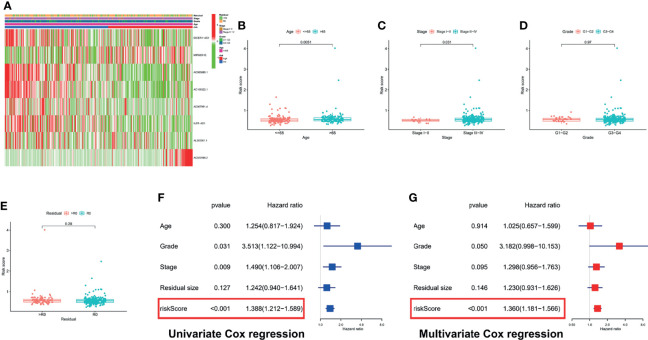
Correlation analysis and Cox regression analysis of risk score combined with clinical variables. **(A)** A composite heatmap combining clinical information. Correlation analysis based on risk grouping, including age **(B)**, FIGO stage **(C)**, grade **(D)**, and residual size **(E)**. **(F)** Forest plot of univariate Cox regression analysis: correlation between OS and clinicopathological features including signature. **(G)** Forest plot of multivariate Cox regression analysis: correlation between OS and clinicopathological features including signature.

First, we showed the overall distribution of different clinicopathological factors in the high- and low-risk groups *via* the expression of eight PRLs in the form of a heat map, as shown in **Figure 6A**. Next, a correlation analysis was performed between the different risk groups and the corresponding clinical characteristics. As presented in **Figure 6**, increased risk scores were significantly related to multiple factors, including age (**Figure 6B**) and FIGO stage (**Figure 6C**) (p < 0.05). It should also be noted that the different risk groups were not statistically correlated with the following clinical characteristics: grade (**Figure 6D**) or residual size (**Figure 6E**). To investigate the independent prognostic value of the risk signature in OC patients, the univariate Cox analysis revealed that the risk score was a high-risk factor (p < 0.001; **Figure 6F**). Furthermore, a further multivariate Cox analysis showed that only the risk score was independently associated with OS (p < 0.001), implying that the risk signature may be an independent prognostic predictor for OC patients (**Figure 6G**). To further verify the prognostic value of our signature, we performed risk scores for each OC patient in the GSE26193 dataset using the same formula. The results showed that our signatures also had an excellent survival prediction ability in the external dataset, and the OS of the high-risk group was shorter than that of the low-risk group (**Figures S1A, B**). In addition, univariate and multivariate Cox analyses also revealed that risk score was an independent prognostic factor (**Figures S1C, D**).

### 3.5 Survival Analysis of the Clinical Subgroup Based on Risk Signature

**Figure 7:**
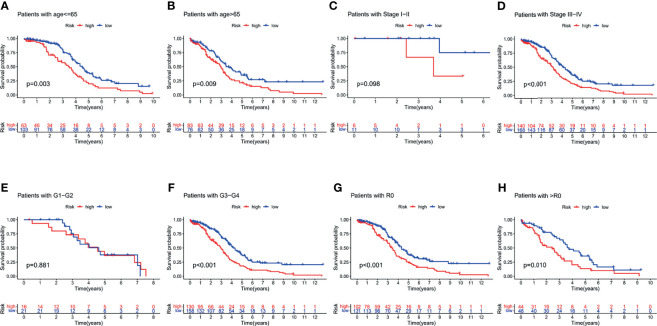
Survival analysis for the clinical subgroup. Survival analysis of subgroups: **(A)** age ≤65 group, **(B)** age >65 group, **(C)** I–II stage group, **(D)** III–IV stage group, **(E)** G1–G2 group, **(F)** G3–G4 group, **(G)** R0 group, and **(H)** non-R0 group.

To further explore the predictive efficiency of the PRL signature on different clinical characteristics from the TCGA cohort, the following clinical variables were used for analysis: age (≤65 and >65 years), FIGO stage (I–II and III–IV), pathological grade (G1–2 and G3–4), and residual tumor size (R0 and non-R0). In the survival analysis of the clinical subgroups, the survival time of the high-risk group was significantly shorter than that of the low-risk group (**Figures 7A, B**), advanced stage group (**Figure 7D**), G3–G4 group (**Figure 7F**), and residual tumor size subgroup (**Figures 7G, H**; *p* < 0.05). In addition, there was no statistical difference in survival in the I–II (**Figure 7C**) and G1–G2 (**Figure 7E**) groups, likely due to the small sample size of early stage patients.

### 3.6 Construction of Two Visual Prognostic Models (Nomograms) Based on Risk Signature

**Figure 8:**
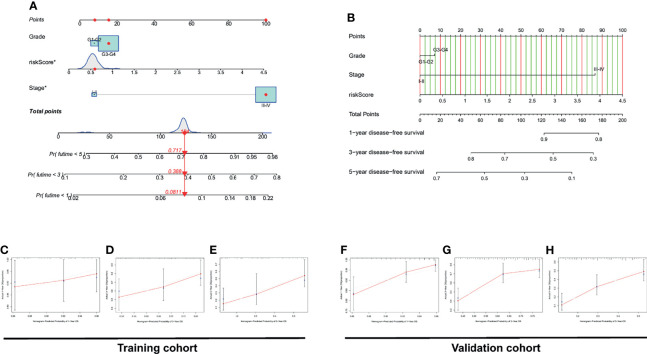
Construction and discrimination analysis of the two nomograms. **(A)** A nomogram plotted by “regplot” package for predicting OS at 1, 3, and 5 years. **(B)** A nomogram plotted by “rms” package for predicting OS at 1, 3, and 5 years. **(C–E)** Calibration curve of the nomogram based on risk signature for OS prediction in the training set. **(F–H)** Calibration curve of the nomogram based on risk signature for OS prediction in the validation set.

Considering that the formula for PRL signatures is complicated, nomograms can intuitively be applied to clinical work; therefore, we visualized the risk signature based on the above risk formula. We combined indicators commonly used in clinical work to construct two visual prognostic models using different packages in R software based on the same risk formula, as shown in **Figures 8A, B**. The calibration curve of the nomogram showed that the prediction curves were close to the standard curve in the two cohorts, which indicates that the predicted survival rate is closely related to the actual rates at 1, 3, and 5 years, as shown in **Figures 8C**–**H**.

### 3.7 Investigation of the Clinical Benefits of Risk Signatures

**Figure 9:**
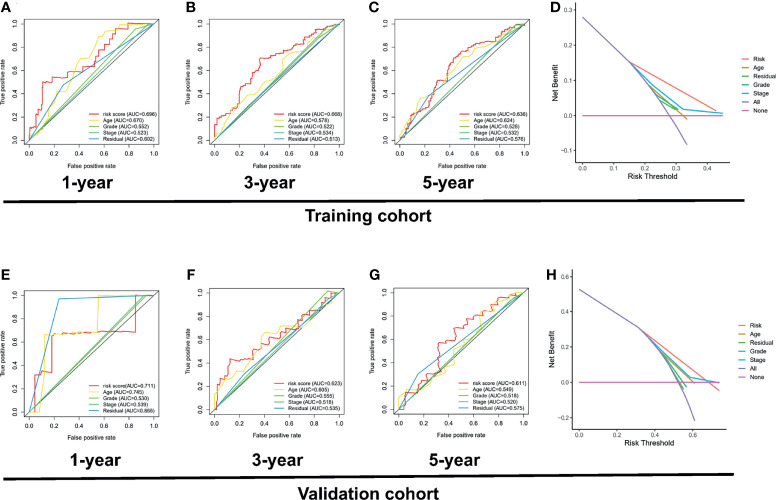
Investigation of the clinical benefit of risk signature using DCA and ROC analysis. ROC curve of clinicopathological features including PRL signature in the training set, including 1 year **(A)**, 3 years **(B)**, and 5 years **(C)**. **(D)** DCA analysis of clinicopathological features including risk signature in the training set. **(E)** ROC curve of clinicopathological features including risk signature in the validation set, including 1 year **(E)**, 3 years **(F)**, and 5 years **(G)**. **(H)** DCA analysis of clinicopathological features including PRL signature in the validation set.

Interestingly, the DCA curve and the ROC curve showed that the PRL signature was significantly better at predicting survival time than traditional clinical characteristics in the training cohort (**Figures 9A**–**D**) and validation cohort (**Figures 9E**–**H**; *p* < 0.05). In addition, to further compare the accuracy of the PRL risk signature, we compared the prognosis signatures of patients with OC in other studies. The results were also exciting: in the C-index for predicting the TCGA cohort, the risk signature of our study showed better predictive value than the glycolysis-related gene signature established by Zhang et al. (34), the glycolysis-related lncRNA signature established by Zheng et al. (35), and the DNA methylation-driven gene signature established by Zhou et al. (36), as shown in **Figure S2**.

### 3.8 The Potential Mechanism of the Eight PRLs

**Figure 10:**
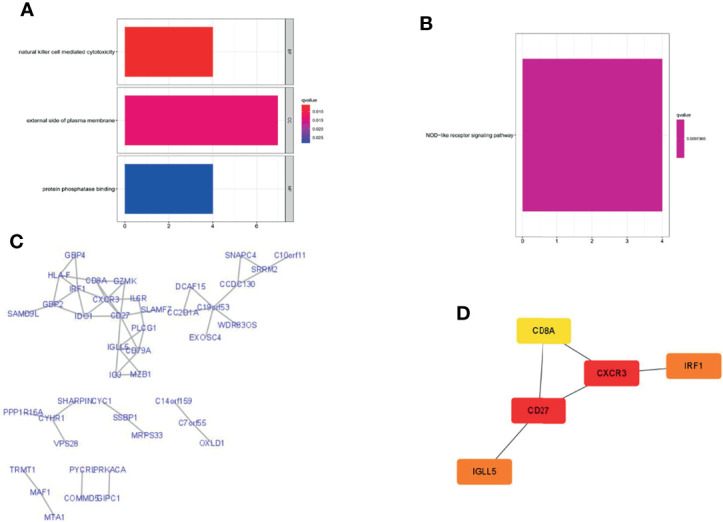
Gene enrichment analysis and PPI network were performed in lncRNA-related genes. **(A)** GO enrichment analysis. **(B)** KEGG enrichment analysis. **(C)** Construction of the PPI network with 80 protein-coding genes. **(D)** Five hub genes in the PPI network.

To explore the potential biological functions and pathways of the eight PRLs in the signature, we screened out genes that were co-expressed with the eight PRLs. Finally, we selected 80 mRNAs based on the cutoff value stated in the *Materials and Methods* section, as shown in **Figure 10C**. GO enrichment analysis (**Figure 10A**) showed that more than 80 mRNAs were mainly related to natural killer cell-mediated cytotoxicity in the BP section, the external side of the plasma membrane in the CC section, and protein phosphatase in the MF section. KEGG enrichment analysis (**Figure 10B**) showed that related mRNAs were enriched in the NOD-like receptor signaling pathway. In addition, we explored the above mRNAs in the PPI network (**Figure 10C**). Finally, as shown in **Figure 10D**, we performed topological analysis of the genes in the PPI network using Cytoscape and identified the top 5 genes: *CD27*, *CD8A*, *CXCR3*, *IRF1*, and *IGLL5*.

### 3.9 Association Analysis Between Risk Signature and *BRCA1/2* Mutations

**Figure 11:**
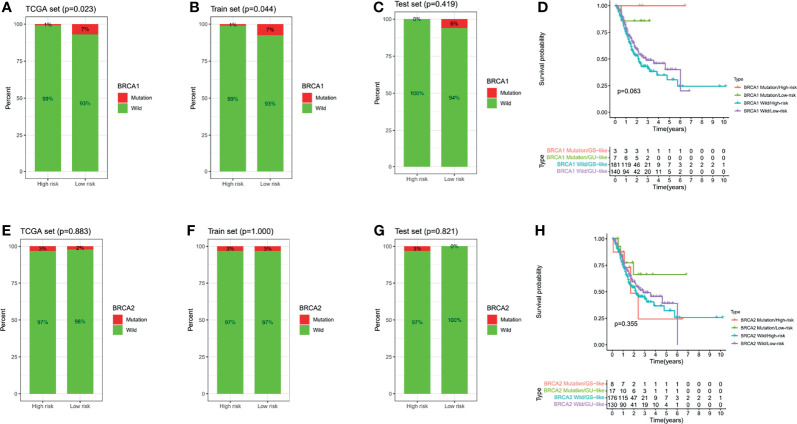
*BRCA1/2* mutations in different risk subgroups with ovarian cancer. Mutations in *BRCA1* among different groups, including the TCGA all set **(A)**, train set **(B)**, and test set **(C)**. **(D)** Survival analysis combining risk score and *BRCA1* mutation. Mutations in *BRCA2* among different groups, including the TCGA all set **(E)**, train set **(F)**, and test set **(G)**. **(H)** Survival analysis combining risk score and *BRCA2* mutation.

Considering the important correlation between the *BRCA1/2* gene in OC patients and maintenance therapy after cytoreductive surgery, we further analyzed the different risk groups for any associations between their risk signatures and *BRCA1/2* gene mutations. According to the mutation data in the TCGA database, we found that there was a statistically significant difference in *BRCA1* gene mutation numbers between the high- and low-risk groups in the TCGA cohort (*p* = 0.023) (**Figure 11A**). However, mutations in this gene were not statistically significant in the TCGA training and testing sets (**Figures 11B, C**). However, in the analysis of *BRCA2* mutations, we did not find significant differences among the groups (**Figures 11E**–**G**). Meanwhile, we combined risk groups with mutations in the *BRCA1/2* genes for survival analysis. The results showed no statistically significant differences between the four groups, as shown in **Figures 11D, H**.

### 3.10 Comprehensive Immune-Infiltration Analysis Based on Risk Signature Subgroups

**Figure 12:**
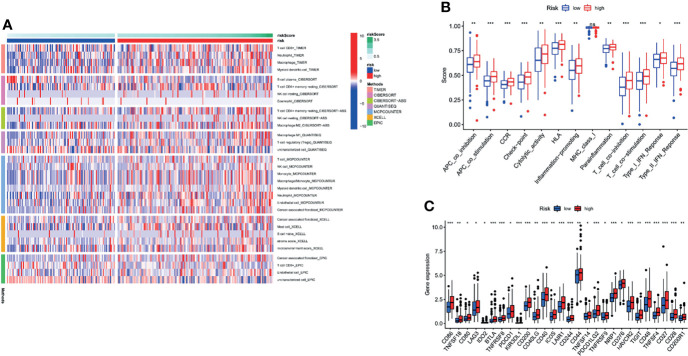
Comprehensive analysis of immune cell infiltration, immune checkpoint, and immune function based on risk signature subgroups. **(A)** A heatmap for different immune cells based on six algorithms. **(B)** Immune function scores in different risk groups. **(C)** Expression of immune checkpoints in different risk groups. *p < 0.05, **p < 0.01, ***p < 0.001, ns, no statistical significance.

To comprehensively explore the relationship between different risk groups and immune cell infiltration, we plotted a heatmap of immune cell infiltration based on six algorithms (TIMER, CIBERSORT, quanTIseq, MCP-counter, xCell, and EPIC), as shown in **Figure 12A**. Interestingly, analysis of immunologic function confirmed significant differences between the low- and high-risk groups for immunological function other than MHC class I (*p* > 0.05), as shown in **Figure 12B**. Finally, it is worth noting that there were significant differences in the expression of some commonly used clinical immune checkpoints between the different risk groups, as shown in **Figure 12C**.

### *3.11 In-Vitro* Assays and Meta-Analysis on Alternative lncRNAs

**Figure 13:**
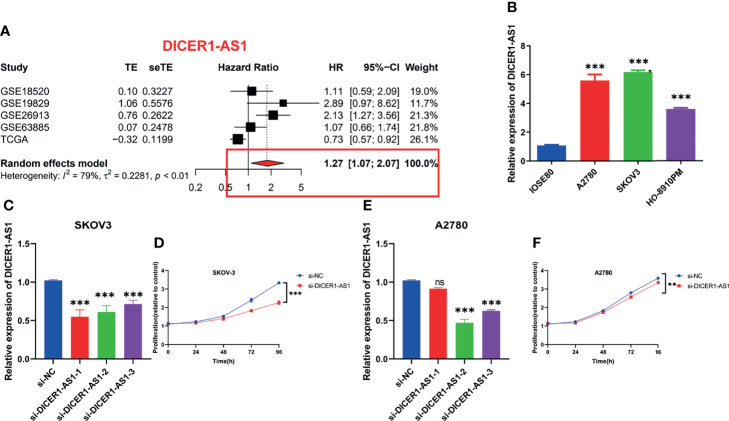
*In-vitro* assays and meta-analysis on alternative lncRNAs. **(A)** Meta-analysis of OS in OC patients about DICER1-AS1. **(B)** Relative expression of DICER1-AS1 in IOSE80, A2780, HO-8910PM, and SKOV-3 cell lines. **(C)** Relative mRNA expression of DICER1-AS1 in SKOV-3 cell lines transfected with si-DICER1-AS1. **(D)** CCK-8 assays in SKOV-3 cell lines transfected with si-DICER1-AS1. **(E)** Relative mRNA expression of DICER1-AS1 in A2780 cell lines transfected with si-DICER1-AS1. **(F)** CCK-8 assays in A2780 cell lines transfected with si-DICER1-AS1. **p < 0.01, ***p < 0.001.

lncRNA *DICER-AS1* was filtered as a candidate molecule in which to perform cell function assays. To further illustrate the prognostic value of *DICER-AS1*, we performed a meta-analysis of *DICER-AS1* by combining several datasets (TCGA, GSE18520, GSE19829, GSE26931, and GSE63885) using a random effects model. Among the results of the meta-analysis of OS, *DICER-AS1* was shown to be a high-risk factor for survival in OC patients [HR = 1.27 (1.07–2.07)], as shown in **Figure 13A**. In three OC cell lines (A2780, SKOV-3, and HO-8910PM), real-time qPCR analysis revealed that DICER-AS1 mRNA expression was substantially upregulated compared with normal ovarian epithelial cells (IOSE80), as shown in **Figure 13B**. We also assessed the efficiency of siRNAs targeting *DICER-AS1* and discovered that siRNA-1 was most efficiently transfected into SKOV-3 cells (**Figure 13C**); in addition, siRNA-2 was most efficiently transfected into A2780 cells (**Figure 13E**). We initially performed CCK-8 experiments, which revealed that the downregulation of *DICER-AS1* expression significantly reduced the proliferation of OC cells (**Figures 13D, F**).

### 3.12 Validation of the Eight lncRNAs

**Figure 14:**
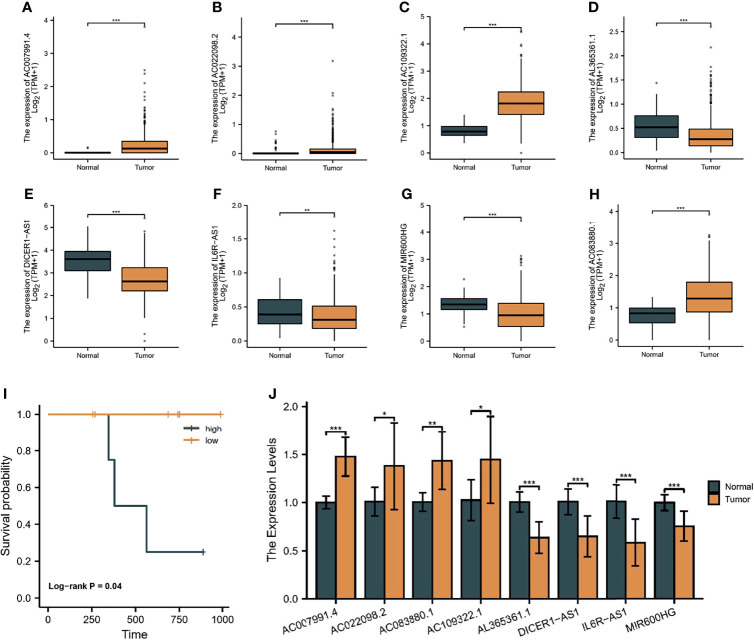
Validation of the eight lncRNAs. The expression of *AC007991.4* **(A)**, *AC022098.2* **(B)**, *AC1093322.1* **(C)**, *AL365536.1* **(D)**, *DICER1-AS1* **(E)**, *IL6R-AS1* **(F)**, *MIR600HG* **(G)**, and *AC083880.1* **(H)** in the TCGA and GTEx databases. **(I)** Survival analysis of our cohort. **(J)** RT-qPCR detection of the expression of eight lncRNAs in 10 pairs of tissues. *p < 0.05, **p < 0.01, ***p < 0.001.

As shown in **Figures 14A**–**H**, we found that four lncRNAs (*AC007991.4*, *AC022098.2*, *AC1093322.1*, and *AC083880.1*) were highly expressed in tumor tissues as compared with normal tissues by differential analysis based on the TCGA and GTEx databases. Meanwhile, *AL365536.1*, *DICER1-AS1*, *IL6R-AS1*, and *MIR600HG* were overexpressed in normal tissues. Notably, *DICER1-AS1* was overexpressed in OC cell lines. Subsequently, RT-qPCR was used to detect the expression of the eight lncRNAs in 10 pairs of tissues. The results were consistent with the prediction results in public databases (**Figure 14J**). Moreover, four patients were considered high risk and six patients were low risk, and their OS rates were also significantly different (**Figure 14I**).

## 4 Discussion

In this study, we detected the overall landscape of PRGs in OC patients, and our results indicate the possibility of pyroptosis pathway activation in OC. In addition, we identified the lncRNAs upstream of these PRLs and constructed an eight lncRNA risk signature using Cox and LASSO regression analyses, which was then validated in a training cohort. Meanwhile, correlative immune algorithms, *BRCA* mutation analysis, and *in-vitro* assays further revealed the importance of the PRL signature in ovarian cancer.

lncRNAs have been proven to play an important role in the occurrence and development of tumors by bioinformatics methods or experiments (37). Meanwhile, pyroptosis is a novel form of cell death that has been shown to be a possible new treatment strategy (3). However, compared with other forms of cell death, there has been relatively little research on pyroptosis and cancer, particularly regarding its specific mechanisms in OC. Our study initially identified eight lncRNAs with functions related to regulating cell pyroptosis and adding prognostic value, providing theoretical support for subsequent studies. We confirmed the role of lncRNA DICER-AS1 in OC cell lines, but we have not yet confirmed whether other lncRNAs also play corresponding roles in the pyroptosis pathway of OC. This question warrants future experimental investigation. At present, only two lncRNAs have been confirmed to regulate genes upstream of pyroptosis-related genes (*NLRP1*) in ovarian cancer. The lncRNAs GAS5 (38) and HOTTIP (39) have been shown to regulate OC cells through *NLRP1* inflammatory vesicle-mediated pyroptosis. Among the eight pyroptosis-related lncRNAs screened, all PRLs, except AC109322.1 and AC022098.2, have been studied in cancer. The sensitivity and specificity of DICER1-AS1 expression in differentiating tumor and non-tumor tissues were 63.3% and 36.7%, respectively (16). Overexpression of MIR600HG inhibits tumor invasion and enhances chemotherapy sensitivity, providing a new strategy for colorectal cancer treatment (40). MIR600HG is involved in the construction of risk signatures for oral squamous cell carcinoma (41) and pancreatic cancer (42). Meng et al. also included AC083880.1 in the prediction of OS in patients with ovarian cancer (43). Interestingly, Zhang et al. used AC007991.4 as one of the three risk signatures for predicting survival in patients with gastric cancer (44). IL6R-AS1 (45) and AL365361.1 (46) also had strong predictive values in patients with different cancers.

Our study has a few limitations that must be clarified in detail. As for the lack of an lncRNA sequencing dataset with survival information for patients with ovarian cancer in the GEO database, the analyses were all performed using the TCGA-OC cohort. In addition, although we performed experimental validation of one lncRNA, more comprehensive *in-vivo* and *in-vitro* experiments are needed to confirm our results.

## 5 Conclusions

We performed a comprehensive and systematic bioinformatic analysis to identify a PRL-related signature. Therefore, the findings of this study are useful for promoting individualized immunotherapy, maintenance treatment, and survival prediction in patients with OC.

## Data Availability Statement

Publicly available datasets were analyzed in this study. Data are available at the TCGA (https://portal.gdc.cancer.gov/) and GEO databases (https://www.ncbi.nlm.nih.gov/geo/).

## Ethics Statement

The studies involving human participants were reviewed and approved by the Ethical Committee of Affiliated Hospital of Nantong University. The patients/participants provided their written informed consent to participate in this study.

## Author Contributions

XC and MS conceived and designed the study. XC, QZ, YZ, and XH were responsible for the materials. XC, QZ, and MS drafted the manuscript. MS and JB critically revised the manuscript. All authors approved the submitted version.

## Funding

This work was supported by the Nantong Applied Research Project (MS12015006) and a Chinese National Scientific Project Grant (30801226).

## Conflict of Interest

The authors declare that the research was conducted in the absence of any commercial or financial relationships that could be construed as a potential conflict of interest.

## Publisher’s Note

All claims expressed in this article are solely those of the authors and do not necessarily represent those of their affiliated organizations, or those of the publisher, the editors and the reviewers. Any product that may be evaluated in this article, or claim that may be made by its manufacturer, is not guaranteed or endorsed by the publisher.

## Supplementary Material

Supplementary Figure 1 External validation in GEO database. **(A)** Survival analysis; **(B)** ROC curve of 1 year,3 years, and 5 years. **(C)** Forest plot of univariate Cox regression analysis; **(D)** Forest plot of multivariate Cox regression analysis.

Supplementary Figure 2 C-index of different signatures
