# Supplementary figures and images for: Derivation, Comprehensive Analysis, and Assay Validation of a Pyroptosis-Related lncRNA Prognostic Signature in Patients With Ovarian Cancer
Source: Front Oncol. 2022 Feb 24;12:780950. doi: 10.3389/fonc.2022.780950 (PMC8912994; doi:10.3389/fonc.2022.780950)

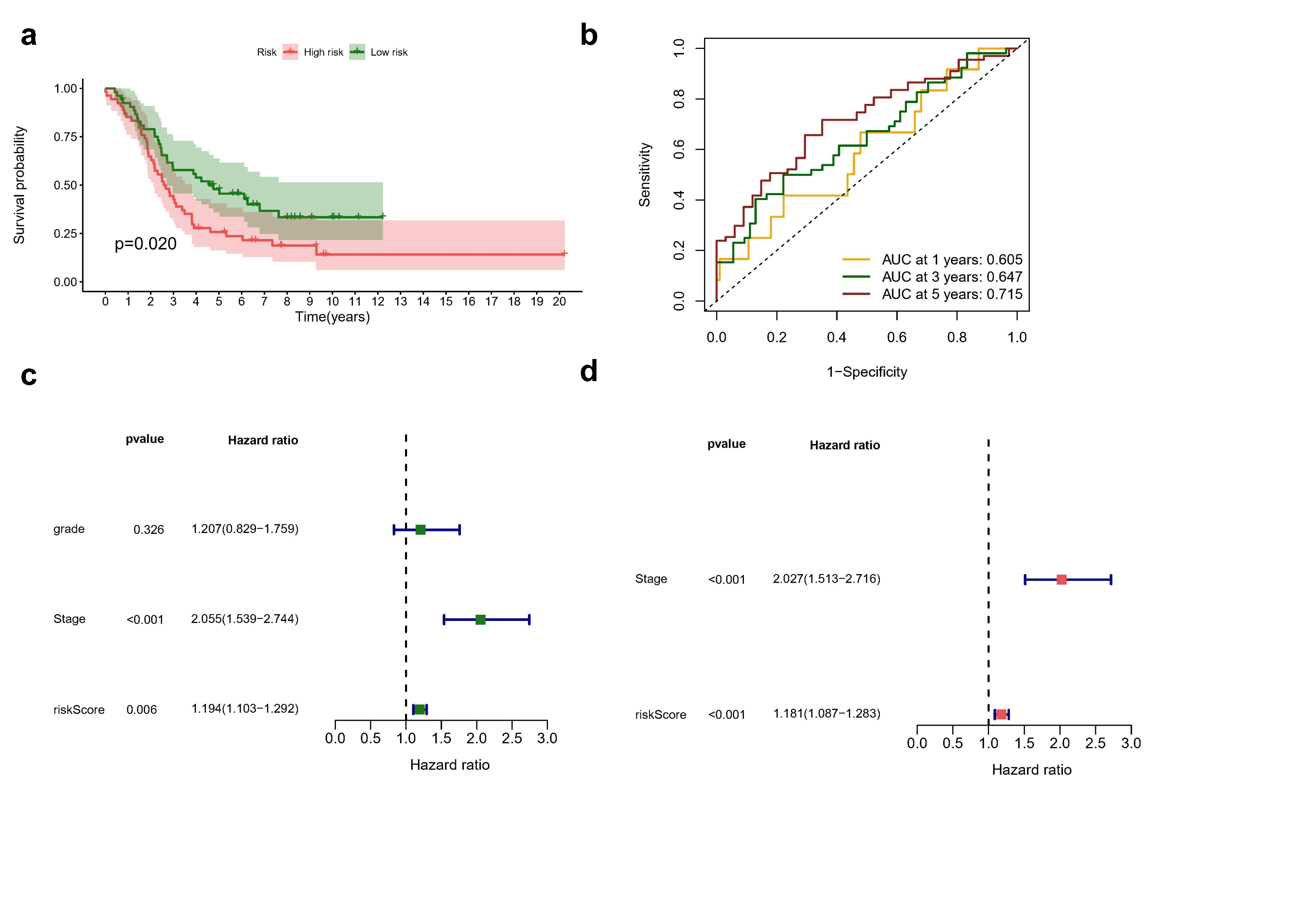

Supplement: Supplementary Figure 1 — External validation in GEO database. (A) Survival analysis; (B) ROC curve of 1 year,3 years, and 5 years. (C) Forest plot of univariate Cox regression analysis; (D) Forest plot of multivariate Cox regression analysis. [file Image_1.tiff]

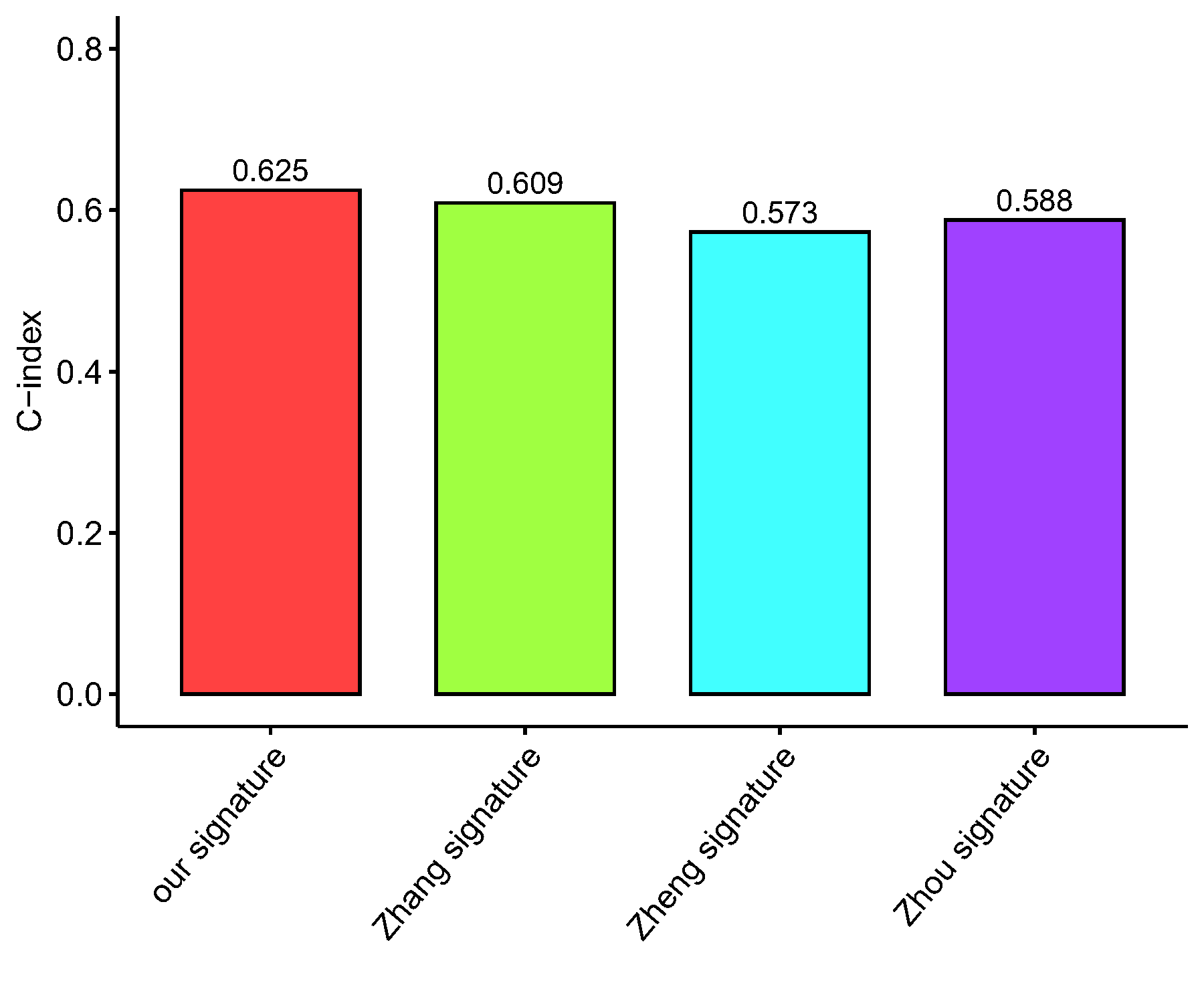

Supplement: Supplementary Figure 2 — C-index of different signatures [file Image_2.tiff]
